# Intervertebral Disc Degeneration Induced by Needle Puncture and Ovariectomy: A Rat Coccygeal Model

**DOI:** 10.1155/2021/5510124

**Published:** 2021-05-17

**Authors:** Tao Tian, Haidong Wang, Zhaohui Li, Sidong Yang, Wenyuan Ding

**Affiliations:** Department of Spine Surgery, The Third Hospital of Hebei Medical University, Shijiazhuang 050051, China

## Abstract

**Objectives:**

To establish a novel animal model of intervertebral disc degeneration (IVDD) in rats and to investigate the effect of 17*β*-estradiol (E2) intervention in this model.

**Methods:**

This study was divided into two parts: animal model (four groups: Sham, ovariectomy (OVX), Puncture, and OVX+Puncture; three-time points: 4, 8, and 12 weeks; three female rats/group/time point) and drug intervention (Sham, OVX+Puncture+corn oil, and OVX+Puncture+E2; three female rats/group). The rats were analyzed by micromagnetic resonance imaging (MRI), hematoxylin and eosin (HE) staining, and safranin-O staining.

**Results:**

MRI and histological scores significantly differed among the four groups at the three-time points (all *P* < 0.05). IVDD progressed with time in the OVX, Puncture, and OVX+Puncture groups (all *P* < 0.05). The changes were the most obvious in the OVX+Puncture group. In the E2 intervention part, the Veh group had the worst MRI signals and histological scores (*P* < 0.05). The MRI scores in the E2 group were less obvious compared to the Sham group (*P* > 0.05). Also, the histological scores were significantly different between the Sham and E2 groups (*P* < 0.05).

**Conclusions:**

The combination of ovariectomy and needle puncture can synergically induce IVDD in rat coccygeal discs. Estrogen treatment can effectively ameliorate IVDD progression in the newly established IVDD models.

## 1. Introduction

Intervertebral disc (IVD) degeneration (IVDD) is a common skeletal condition in humans that is associated with a huge burden on personal and social life [1, 2]. The estimated lifetime prevalence of low back pain (LBP) is 80% [2], and an intervertebral disc is often the source of such back pain [1–3]. The high-risk factors include age, sex, smoking, occupational exposure, body mass index, and family history [2].

While the exact pathogenesis of IVDD is poorly understood, decreases in the levels of estradiol (E2) have been associated with IVDD [4]. The effect of estrogen on the spine has been found in humans and verified in primates. Morenko et al. [5] reported that in rhesus monkeys, scoliosis appeared within 1 year after therapeutic bilateral ovariectomy in the treatment of endometriosis and that the curvature of the spine showed a progressive trend over the next 8 years. In addition, the prevalence of LBP in women is higher than that in men, and it is more obvious after menopause [6, 7].

Abnormal stress may be a triggering condition for IVDD. It can cause slight damage to the IVD and changes in the extracellular matrix (ECM), which in turn cause endplate degeneration and degeneration. Cell senescence or apoptosis can also lead to changes in inflammatory factors, upregulation of related genes, and activation of cell pathways [2, 8, 9]. Changes such as endplate fractures or bone fractures reduce IVD nutrient supply, which causes neoangiogenesis in the IVD, thus leading to the occurrence of immune reactions [2, 8, 9]. Once degeneration starts, the contents of proteoglycans (PG) and collagens reduce, and the nucleus pulposus (NP) loses water and shrinks, which in turn leads to changes in the biomechanical function of IVD neoangiogenesis.

Therefore, the prevention and treatment of IVDD should be comprehensive and include many different aspects [1–3]. On the one hand, correcting living habits, such as sitting for a long time and reducing abnormal stress stimulation, are essential in preventing IVDD. In addition, drugs can be used to control inflammation and pain [1–3]. Nevertheless, the effectiveness of such methods is limited. Currently, various cytokines, biomimetic NP tissue materials, heparin-functionalized collagen gel, and stem cells are being explored [10–12].

Over recent years, basic research related to IVD biology and tissue engineering has gradually emerged in major medical centers, including cell transplantation, cytokine injection therapy, supplementation of biologically active substances, gene therapy, and biomimetic materials. Nevertheless, the molecular biological mechanisms causing IVDD are still unclear. Unfortunately, the animal models of IVDD have several limitations in translation from and to humans [13, 14]. Therefore, this study is aimed at establishing a novel animal model of IVDD in rats and examining the effect of estradiol intervention in this model. Our results could provide additional data on the pathogenesis of IVDD as well as simple methods to manage it.

## 2. Materials and Methods

### 2.1. Study Design and Animals

This study was divided into two parts: the animal model (four groups: Sham, ovariectomy (OVX), Puncture, and OVX+Puncture; three-time points: 4, 8, and 12 weeks; three rats/group/time point: 36 rats in total) and drug intervention (Sham; OVX+Puncture+corn oil, and OVX+Puncture+E2; three rats/group, nine rats total). The animals were female Sprague-Dawley rats (3 months of age (weighting 300 ± 18 g)) acquired from the Laboratory Animal Center of Jilin University (Changchun, China). The rats were randomly assigned to each group. In the Sham groups, only the skin incision was performed. In the OVX groups, the bilateral ovaries were removed. In the Puncture groups, puncture of the IVD of the tail was performed.

### 2.2. Ethics

All animal experiments were performed in accordance with the Guidelines for the Care and Use of Laboratory Animals. The study was approved by the Institutional Animal Ethics Committee of Hebei Medical University (Ethical approval number: Z2020-002-1).

### 2.3. Animal Model Establishment

For the OVX group, the rats were anesthetized using sodium pentobarbital (30 mg/kg, intraperitoneally) and placed in the lateral position. After the skin was prepared and disinfected with iodophor, a 1 cm incision was made under the ribs. The layers were cut open to reach the ovaries. A knot was made at the root of the ovary, the ovary was removed with scissors after ligation, and the incision was sutured. The ovariectomy on the other side was carried out in the same way.

For the Puncture group, the rats were anesthetized and placed in the supine position. The tail was disinfected with iodophor. The skin was incised to the deep fascia layer, and three segments (Co5-6, Co6-7, and Co7-8) were selected. After accurate positioning, a 21 G needle was inserted into the IVD about 3 mm; the needle was rotated for 360° and kept for 30 s, after which the needle was pulled out, and the skin was sutured. Micro-MRI and histological evaluation were performed on randomly selected 3 rats after 4, 8, and 12 weeks, respectively.

### 2.4. Drug Intervention

E2 (#E2758, Sigma, St. Louis, MO, USA) was dissolved in corn oil (#HY-Y1888, MCE, Ohio, US), shaken at room temperature for 10 h, and then stored at 4°C. In the Sham group, only skin incision and suture were performed. The Veh group underwent bilateral OVX and Puncture of the tail IVD. After the operation, corn oil was subcutaneously injected every day. The E2 group underwent bilateral OVX and Puncture of the tail IVD, and E2 oil (10ug/kg) was subcutaneously injected every day after the operation. Micro-MRI and histological evaluation were performed after 12 weeks.

### 2.5. Micromagnetic Resonance Imaging (MRI) Detection

After successful inhalation of isoflurane anesthesia in rats, a micro-MRI system was used to scan the intervertebral disc (sagittal plane) of the rat's tail. The imaging sequence was a self-selected dial-back T2-weighted image used to obtain fat-free scanning parameters: enrichment time: 3000 ms; response time: 80 ms; field of view: 200 × 200 mm; slice thickness: 1.4 mm. The MRI signal evaluation was performed according to the standards by Sobajima et al. [15, 16]. All images were jointly observed and evaluated by three authors using the Pfirrmann grading system [17]. The data was the average of the results obtained by the three evaluators and expressed as the magnetic resonance index.

### 2.6. Histological Evaluation

The three punctured segment vertebral bodies were fixed in 4% paraformaldehyde for 24 h, decalcified using 10% EDTA for 4 weeks, and processed for paraffin embedding. Consecutive 4 *μ*m thick sections of the IVD were made using a microtome. The sections were stained with HE (blue nucleus and red cytoplasm) and Safranin-O (red or orange-red cartilage, green collagen). After the images were collected, they were all evaluated by three observers using Histologic Grading Scores [18]. The data was the average of the results obtained by the three observers expressed as the histological score index.

### 2.7. Statistical Analysis

The micro-MRI and histological scores are ordinal data that were analyzed using the rank-sum test in SPSS 24.0 (IBM, Armonk, NY, USA). Two-sided *P* values < 0.05 were considered statistically significant.

## 3. Results

### 3.1. Establishment of the New Animal Model of IVDD

**Figure 1:**
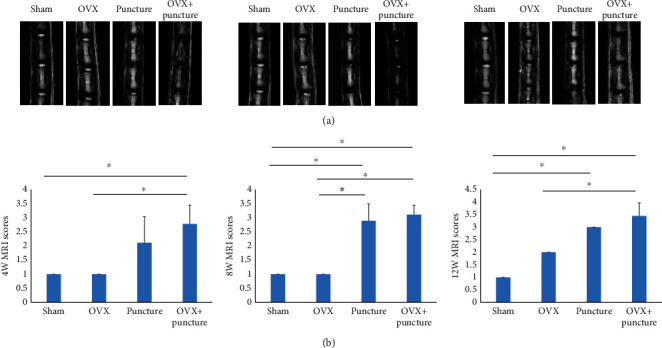
(a) Representative micromagnetic resonance imaging (MRI) of animals in the four groups at 4, 8, and 12 weeks. (b) MRI scores at 4, 8, and 12 weeks. ^∗^*P* < 0.05.

Micro-MRI of the intervertebral disc showed that the NP in the Sham group was full, without obvious morphology and signal level changes. The NP of the OVX, Puncture, and OVX+Puncture groups showed varying degrees of damage and signal levels at 4, 8, and 12 weeks. At 4 W, there was a significant difference between the Sham group and the OVX+Puncture group, between the OVX group and the OVX+Puncture group (*P* < 0.05), and between the Sham group and the OVX group, between the Sham group and the Puncture group, and the OVX group. There was no significant difference between the Puncture group and the OVX+Puncture group (*P* > 0.05). At 8 W, there were significant differences between the OVX group and the OVX+Puncture group (*P* < 0.05), while there was no significant difference between the Sham group and the OVX group, and the Puncture group and the OVX+Puncture group (*P* > 0.05). At 12 W, there were significant differences between the Sham group and Puncture group, between the Sham group and OVX+Puncture group, between the OVX group and OVX+Puncture group (*P* < 0.05), while there was no significant difference between the Puncture group and the OVX+Puncture group (*P* > 0.05) (Figure 1).

Considering time points, there were significant differences between 4 and 12 weeks and between 8 and 12 weeks in the OVX group (*P* < 0.05). The difference between 4 and 12 weeks in the Puncture group was significant (*P* < 0.05). The difference between 4 and 12 weeks in the OVX+Puncture group was significant (*P* < 0.05). By comparison, we found that the degree of degeneration of the intervertebral discs in the three groups was positively correlated with the prolongation of time (Figure 1).

### 3.2. Histological Analysis of the Rat Model of IVDD

**Figure 2:**
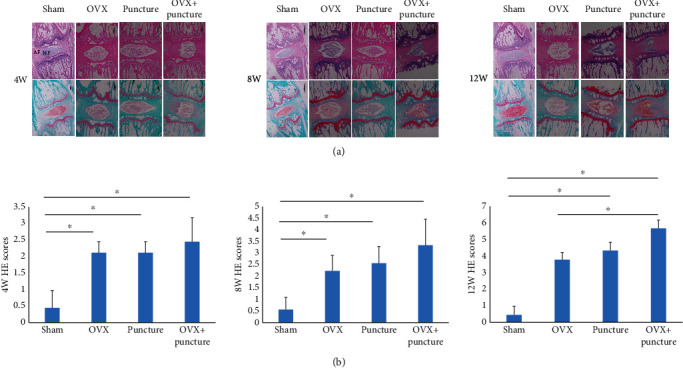
(a) Hematoxylin and eosin (HE) staining and safranin staining of discs in the four groups at 4, 8, and 12 weeks. (b) HE scores at 4, 8, and 12 weeks. ^∗^*P* < 0.05.

HE staining was applied to further evaluate the rat model.  Figure 2 showed that the AF ring in the Sham group was not significantly distorted, and the number of NP cells was normal. In the OVX, Puncture, and OVX+Puncture groups, there was a distortion of the AF ring, and the number of NP cells was greatly reduced. At 4 W, the differences between the Sham group and the OVX group, between the Sham group and the Puncture group, and between the Sham group and the OVX+Puncture group were significant (*P* < 0.05), while there was no significant difference between the Puncture group and the OVX+Puncture group (*P* > 0.05). At 8 W, the difference between the OVX group and the Puncture group was significant (*P* < 0.05), and there was no significant difference between the OVX group and the Puncture group, between the OVX group and the OVX+Puncture group, and between the Puncture group and the OVX+Puncture group (*P* > 0.05). At 12 W, there were significant differences between the Sham group and Puncture group, between the Sham group and OVX+Puncture group, and between the OVX group and OVX+Puncture group (*P* < 0.05), while there was no significant difference between the Puncture group and the OVX+Puncture group (*P* > 0.05).

Furthermore, in the OVX group, there were significant differences between 4 and 12 weeks and between 8 and 12 weeks (*P* < 0.05). In the Puncture group, there were significant differences between 4 and 12 weeks and between 4 and 8 weeks (*P* < 0.05). In the OVX+Puncture group, there were significant differences between 4 and 12 weeks and between 8 and 12 weeks (*P* < 0.05). The degree of degeneration of the IVD in the three groups was correlated with time (Figure 2).

Taken together, these results showed that using OVX, Puncture separately, or OVX+Puncture could all achieve IVDD, where the changes in OVX+Puncture models were the most noticeable. Compared to OVX, OVX+Puncture demonstrated significant differences, while compared to Puncture, there might be discrepancies after 12 w.

### 3.3. Effect of E2 on IVDD in the Rat Model

**Figure 3:**
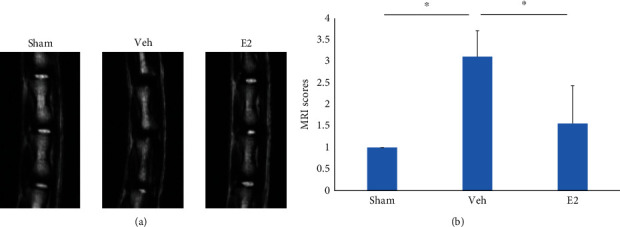
(a) After successful modeling, the Sham, Veh, and E2 groups underwent micromagnetic resonance imaging (MRI). (b) MRI scores ^∗^*P* < 0.05.

Next, based on OVX+Puncture rat models, we evaluated the effect of E2 treatment in IVDD. After 12 weeks of E2 intervention, micro-MRI detection revealed that the disc morphology and T2 signal of the Veh and E2 groups changed compared with the Sham group. The T2-weighted signal of the micro-MRI in the Veh group was significantly weaker than that of both Sham and E2 groups, and grade IV IVDD was observed. The changes in the E2 group were less obvious than in the Sham group (*P* > 0.05). There were significant differences between the Sham group and the Veh group (*P* < 0.05) and between the E2 group and the Veh group (*P* < 0.05) (Figure 3).

**Figure 4:**
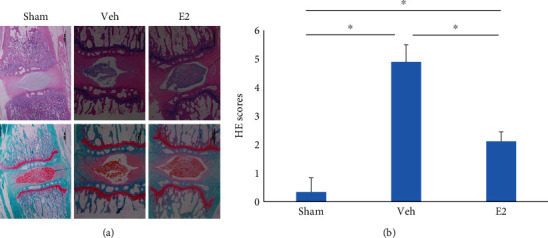
(a) Hematoxylin and eosin (HE) staining and safranin staining of discs in the three groups. (b) HE scores. ^∗^*P* < 0.05.

The HE staining results showed that in the Veh group, the AF ring was distorted, and the number of NP cells greatly decreased. The shape of the AF ring and the number of NP cells in the E2 group were similar to that in the Sham group. Significant differences were found between the Sham and Veh groups (*P* < 0.05), between the E2 and Veh groups (*P* < 0.05), and between the Sham and E2 groups (*P* < 0.05) (Figure 4). In summary, these results showed that E2 has a role in protecting the IVD against degeneration.

## 4. Discussion

Low back pain (LBP) is a common human disease, which brings a huge economic burden to individuals and society. Consequently, the research on this disease is of great significance. The oldest known written record dates back to around 1500 BC. Hippocrates (460 BC-370 BC) was the first person to use the terms sciatic nerve pain and low back pain, while Galen described this concept in detail. For a long time, doctors suggested using conservative medieval treatment. It was not until the beginning of the twentieth century that people gradually understood the cause and pathological process of LBP. With the use of X-ray, CT, MRI, and other technologies in clinical practice, people gradually realized that the intervertebral disc might be the source of back pain and tried to relieve the pain symptoms through surgery. The structure of the human intervertebral disc is very complex, and no animal model can perfectly replicate this structure. Intervertebral disc degeneration is a complex process, and the currently used IVDD animal model has many limitations [13, 14]. Accordingly, establishing an efficient animal model is very important for understanding this pathological process and finding potential treatment methods.

The production of animal models should meet the following requirements: (1) animals should be of sufficient size to allow the direct introduction of therapeutic agents into the intervertebral discs, and the anatomical and physiological characteristics of the selected animals should be as similar to humans as possible; (2) low-priced animals with relatively high repeatability and reliability; (3) IVDD that develops in a gradual time-dependent manner; (4) IVDD histologically close to spontaneous degeneration that can reproduce the objective law of degenerative disc disease and truly simulate the process of human disc degeneration. Many experimentally induced IVDD animal models have been used in preclinical research and are usually divided into mechanical, physical, and chemical models. Although the used animals such as rabbits or dogs are large enough in most mechanical models, these models have disadvantages such as high price and moral controversy. Physical models such as AF injury, AF damage caused by Puncture needles or scalpels, cause the instability of the intervertebral disc complex. Over time, they can better simulate the process of intervertebral disc degeneration. Another commonly used animal model is chemical lysis, in which proteolytic enzymes (hyaluronidase, papain, or chondroitinase ABC) are injected into the nucleus pulposus. These enzymes induce the chemical degradation of the extracellular matrix of the nucleus pulposus, especially proteoglycans and collagen. The main disadvantage is that it induces an acute degenerative process. O'Connell et al. measured the intervertebral disc height, lateral width, front-to-back width, and other parameters of different animals and compared them with the human body. They are sorted according to the similarity with the human body geometry: mouse lumbar spine (12%), rat lumbar spine (15%), mouse tail (18%), baboon (19%), oxtail (22%), rabbit (26%), sheep (31%), and rat tail (46%) [19]. It is not difficult to see that the rat tail is an ideal research object. Efficient animal models require the simulation of human pathological changes in the shortest possible time. In their animal experiments, Deng et al. confirmed that lumbar disc degeneration occurred in female rats at 6 months after ovariectomy [20]. Due to the long experimental period, it is difficult to meet the experimental requirements. Also, with the extension of the test time, the mortality of animals gradually increases; thus, researchers continue to seek new methods to shorten the modeling time. This study is aimed at establishing a novel animal model of IVDD and examining the effect of estradiol intervention in such a model. Our results showed that the IVDD model established with OVX+Puncture of the IVD makes it easier to establish IVDD, and the degree of degeneration based on MRI and histological analysis is relatively more severe compared with OVX or Puncture separately. E2 treatment alleviated the IVDD in OVX+Puncture rat models.

Many studies have shown that the process of IVDD has close relevance to the spontaneous generation of considerable amounts of cytokines [2, 8, 9, 11, 21]. Disc Puncture is one method used to induce IVDD in animals [13, 14]. E2 deprivation or lower levels of estrogens have been associated with IVDD [4–7, 22]. Therefore, in this study, a rat model combining both IVD Puncture and E2 deprivation was established. It has been reported that estrogen can reduce the apoptosis of IVD cells in a variety of ways [23]. Moreover, E2 can inhibit apoptosis of rat nucleus pulposus cells by downregulation of MMP-3 and MMP-13 [24, 25]. Guo et al. [26] found that E2 could protect against apoptosis in human NP cells in vitro. Jin et al. [27] showed that E2 supplementation increased the expression of enzymes involved in the protection against oxidative stress and inhibited autophagy. In addition, estrogen deprivation has been associated with remodeling of the endplates that influence the conditions of the IVD [28]. In this study, the OVX+Puncture group showed the most obvious response to MRI and histological degeneration. Furthermore, supplementing E2 to OVX+Puncture rats prevented disc degeneration. These results suggest that E2 could be used as a method to prevent or manage IVDD in selected patients.

The present study has some limitations. The appropriate concentration range and the action time of E2 that protected against IVDD were not explored in this study. Further studies are needed to closely examine the involved proteins and mRNA.

## 5. Conclusions

A coccygeal IVDD model can be successfully established in female rats by the combination of ovariectomy and needle puncture to the IVD. Estrogen can protect against IVDD progression in the newly established models.

## Data Availability

The datasets used and/or analyzed during the current study are available from the corresponding author on reasonable request.
